# Supplementary material for: Who can go back to work when the COVID-19 pandemic remits?
Source: PLoS One. 2020 Aug 27;15(8):e0238299. doi: 10.1371/journal.pone.0238299 (PMC7451540; doi:10.1371/journal.pone.0238299)
Supplement: S1 Appendix — (DOCX) [file pone.0238299.s001.docx]

Appendix 1

Notes about the situation in Spain.

**A.- Lockdown**

In Spain, one of the countries most severely affected by the pandemic, a strict lockdown has been imposed in economic terms. It has been applied nationwide by establishing three types of activities: essential activities (not affected by lockdown), prohibited activities, and activities not included in either of the other categories (Royal Decree 463/2020, March 14). When the virus was at its most propagative, this latter group of activities was also brought to a standstill for two weeks by establishing compulsory paid rest period for workers, between March 30 and April 9, which would then involve the hours lost being recovered later in the year (Royal Decree-law 10/2020, March 29).

The economic activities forbidden were all of those involving social contact with people and have mainly affected the following sectors: hotel and catering, commerce, leisure, culture, sport (Royal Decree 463/2020, March 14 (art. 10)). Later, tourism, international passenger transport and accommodation were also added (Order SND/257/2020, March 19). In addition, face-to-face teaching was immediately prohibited although teaching did continue because the government imposed teleworking on teachers and other education staff (Royal Decree 463/2020, March 14 (art. 9)). These measures have been accompanied by unemployment benefits and subsidies to individuals and companies in order to partially compensate for the reduction or loss of income. Loans, public guarantees and deferred payment obligations, including tax obligations, have also been implemented in order to reduce cash flow stress (Royal Decree-law 8/2020, March 17 and Royal Decree-law 15/2020, April 21).

In the labour field, the government did not initially prohibit dismissal, which resulted in the dismissal of many casual workers. Finally, it was prohibited under Royal Decree-law 9/2020, March 27. As an alternative, the government facilitated the implementation of collective dismissals on a temporary basis – furloughing - (Spanish acronym - ERTE) and established economic compensation for self-employed workers (appendix of Royal Decree 463/2020 and Royal Decree-law 11/2020, March 31).

**B.- Lockdown exit strategy**

The following is a brief description of the lockdown exit procedure established by the Spanish government^32^. As we can see, it fails to specify the number of workers who will be allowed to return to work, which is the problem we aim to address in this document.

**Health criteria for the lockdown de-escalation strategy**

At the end of April, at the time of writing this paper, the Spanish government began to define the health criteria and markers for the lockdown de-escalation strategy to be applied throughout May, for a progressive, gradual and asymmetric exit from lockdown. There would basically be four markers out of the 27 requirements proposed: infection rate per each infected person, accumulated incidence of cases, hospital rate, and percentage of ICU occupancy. The technical proposal (Centre for Coordination of Health Alerts and Emergencies –CCAES, 2020) is that the lockdown exit strategy and the gradual return to a new normality should be slow and complex and should begin when:

- An area has had an infection rate of less than 1 (basic reproduction number R_0_) for at least two weeks. This marker indicates that each patient has infected less than one other person. According to data, in Spain the average at this precise moment is exactly one. So far, there are fourteen regions below this indicator, with Murcia, Extremadura, La Rioja and Asturias being the best placed.
- The incidence of new cases per day is 2 per 100,000 inhabitants: that is, about 1,000 new cases every day. The issue is that now over 4,000 cases are reported although both new and old cases are being included; i.e. people who test positive but who have already overcome the disease. With the latest data offered, the Balearic Islands, the Canary Islands, and Murcia would meet the requirements, while Andalusia, Extremadura, and Asturias would be close to doing so.
- The area must have sufficient health care capacity available, should there be a fresh outbreak or second wave (as happened a century ago with the Spanish flu of 1918) in order to avoid pushing health care capacity to the limit. The procedure establishes two criteria: the occupation of beds in ICUs for coronavirus patients should be less than 50% and the hospitalization rate of this type of patient should be reduced to 30%. As regards these criteria, not all regions are counting daily admissions but are reporting the accumulated total instead. Although we do not have data from both indices, several regions have ICUs vacated above 50%, and there are many that are close to meeting the maximum hospitalization rate of 30% of patients with coronavirus.

Another aspect to consider is that hospitals must have the means to maintain a dual entry circuit in health centres so as to prevent the spread of the virus and must have the capacity to perform PCR tests on all patients admitted to hospitals. As regards primary care, health centre capacity to detect COVID-19 cases early must be guaranteed and they must have the capacity to isolate any cases that arise, as well as monitor contacts epidemiologically. To this end, there must be specific centres to care for patients with suspected COVID-19 symptoms. In addition, basic protective measures such as maintaining safe distances, frequent hand-washing and the use of face masks should be fostered.

**Social, economic and temporal criteria for the lockdown de-escalation strategy**

On April 28, 2020 the Spanish government approved the national plan to progressively put an end to the lockdown measures in the following months. This plan "for a transition to the new normality" is based on four different temporary phases, each of which lasts approximately two weeks. In addition, the implementation of each phase will be based on the specific situation of each province, i.e. the plan has a geographical dimension.

This forecast is indicative rather than exhaustive. The decisions and specific dates on the effective lifting of the limitations established during the state of emergency will be determined by the corresponding legal instruments. The common regime applicable to all measures may also be adapted, depending on the evolution of the pandemic or other justified circumstances.

Geographical asymmetry does not mean that lockdown de-escalation will be full-scale by areas. Rather, what it does is to classify areas according to health criteria and, taking into account this situation, areas will evolve with a selective gradual lockdown de-escalation strategy by sectors of activity:

- PHASE 0 (May 4, 2020): opening of small shops and businesses by appointment for individual attention. For example, restaurants will offer a food delivery service. There will be individual training for federated athletes and professional leagues. There will also be signage of public places in order to ensure social distancing is maintained. Bathroom facilities must provide maximum protection. Face masks will become compulsory on public transport. There will be no interprovincial mobility.
- PHASE 1 (May 11, 2020): possibility of moving within the same province. Activities will begin in small shops "under strict safety conditions ", but not in large stores "where crowds are more likely". Street cafés and terraces will be allowed to open at 30% of their capacity, without any inflow of customers inside the premises. Opening of hotels and other tourist accommodation, excluding common areas and with a preferential schedule for people over 65 years of age. Agri-food and fishing activities that had been stopped under the decree issuing the state of emergency will resume. Places of worship can open but are limited to one third of their capacity. Professional athletes will have fewer restrictions, since “normal training” will be authorized in professional leagues and in high-performance centres. Hygiene measures and reinforced protection. Face masks will become compulsory on public transport.
- PHASE 2 (approx. May 25, 2020): the inner areas of bars and restaurants will be opened with a third of their capacity, provided there are “guarantees of separation and only for table service”. The school year will start in September, but in this phase, exceptions are established to reopen educational centres. They may open for reinforcement activities, so as to ensure that children under the age of six can go to the school if both parents must work on site. School will open for university entrance examinations. Cinemas, theatres, concert halls, and other similar spaces will reopen with "preassigned seats" and seating will be limited to one third of the usual capacity. People will also be able to visit monuments and exhibition halls, with the same restrictions on capacity. In addition, cultural shows such as concerts can be held in closed spaces, with one third of the usual capacity. If they are outdoors, up to 400 people will be allowed to congregate if they are seated. The capacity of places of worship is limited to 50%, as opposed to the one third of the previous phase. Hunting and sport fishing will resume. Face masks are compulsory on public transport.
- PHASE 3 (approx. June 8, 2020): general mobility will be made more flexible and, although it is the least restrictive phase, the use of face masks on public transport will remain compulsory, as in all previous phases. Shops: capacity will be limited to 50%, with the requirement that there be a minimum distance of two metres between people. Measures in the catering sector will be relaxed, although strict measures regarding separation between the public will be maintained.

Any permitted activity must be carried out in conditions of safety, self-protection, and ensuring social distancing. The use of masks outside the home is advisable and strongly recommended when social distancing cannot be guaranteed. Likewise, the availability of and access to hydroalcoholic solutions should be provided so as to allow for frequent hand hygiene.
