# Supplementary material for: Who can go back to work when the COVID-19 pandemic remits?
Source: PLoS One. 2020 Aug 27;15(8):e0238299. doi: 10.1371/journal.pone.0238299 (PMC7451540; doi:10.1371/journal.pone.0238299)
Supplement: S2 Appendix — (DOCX) [file pone.0238299.s002.docx]

Appendix 2

Affiliated workers by province

**Table A2.1 Social security affiliated workers by activity group according to the National Classification of Economic Activities 2009, CNAE 2009, by province (February 29, 2020).**

| ***AACC*** | ***Province*** | ***FarmLivesForstFishA*** | ***ExtractiveIndB*** | ***ManufactIndC*** | ***EnergySupD*** | ***WasteWaterSupE*** | ***ConstructionF*** | ***CommerceVehiclRepG*** | ***TransportStorageH*** | ***Hotels…***  ***I*** | ***InformatCommunicatJ*** | ***FinancActInsurancesK*** | ***RealStateActL*** | ***ProfActvScientTechM*** | ***AdmtActvAuxilServN*** | ***PubAdminDefenSSO*** | ***EducationP*** | ***SanitActvSocialServQ*** | ***ArtisActvRecEntrR*** | ***OtherServicesS*** | ***HousehDomestST*** | ***ExtraterritoriaOrgU*** | ***TOTAL*** |
| --- | --- | --- | --- | --- | --- | --- | --- | --- | --- | --- | --- | --- | --- | --- | --- | --- | --- | --- | --- | --- | --- | --- | --- |
| *ANDALUSIA* | *Almeria* | 78,042 | 572 | 12,992 | 394 | 2,217 | 15,623 | 68,778 | 17,193 | 21,171 | 2,493 | 4,621 | 1,469 | 8,419 | 11,290 | 13,008 | 12,784 | 21,599 | 3,318 | 5,622 | 2,924 | 11 | 304,540 |
|  | *Cadiz* | 34,594 | 278 | 31,361 | 604 | 4,592 | 25,567 | 65,473 | 15,632 | 41,503 | 3,634 | 4,842 | 2,507 | 12,719 | 23,718 | 25,103 | 22,750 | 38,010 | 6,415 | 11,472 | 5,024 | 19 | 375,817 |
|  | *Cordoba* | 68,803 | 169 | 31,442 | 608 | 2,378 | 15,981 | 44,434 | 9,770 | 17,654 | 2,850 | 4,575 | 1,259 | 9,311 | 13,262 | 16,290 | 15,544 | 27,835 | 3,292 | 7,510 | 3,826 | 7 | 296,800 |
|  | *Granada* | 58,066 | 420 | 18,474 | 528 | 2,564 | 18,953 | 57,798 | 13,004 | 30,493 | 5,743 | 5,009 | 2,015 | 13,043 | 18,235 | 20,823 | 20,685 | 31,937 | 5,656 | 8,890 | 6,133 | 16 | 338,485 |
|  | *Huelva* | 92,622 | 2,115 | 12,294 | 265 | 1,355 | 10,847 | 27,243 | 8,004 | 15,182 | 1,159 | 2,029 | 701 | 4,664 | 9,445 | 14,185 | 9,026 | 16,615 | 1,905 | 4,127 | 1,506 | 1 | 235,290 |
|  | *Jaen* | 63,164 | 170 | 26,030 | 260 | 1,744 | 11,235 | 31,948 | 7,914 | 13,109 | 1,398 | 3,271 | 444 | 6,662 | 9,617 | 14,836 | 10,365 | 20,051 | 1,953 | 5,091 | 2,240 | 5 | 231,507 |
|  | *Malaga* | 36,605 | 390 | 26,752 | 554 | 6,007 | 54,621 | 112,918 | 25,025 | 79,660 | 14,741 | 10,847 | 9,708 | 31,061 | 49,571 | 29,687 | 32,699 | 52,777 | 12,138 | 20,693 | 15,133 | 130 | 621,717 |
|  | *Sevilla* | 92,376 | 673 | 58,187 | 1,231 | 5,781 | 43,156 | 119,643 | 31,594 | 56,205 | 22,326 | 12,585 | 4,706 | 36,791 | 60,348 | 46,649 | 45,050 | 61,999 | 11,995 | 20,238 | 13,268 | 30 | 744,831 |
| *ARAGON* | *Huesca* | 13,123 | 99 | 13,324 | 258 | 785 | 7,796 | 13,545 | 3,835 | 8,967 | 899 | 1,219 | 358 | 3,112 | 4,847 | 8,068 | 2,855 | 9,582 | 2,526 | 2,629 | 1,488 | 0 | 99,315 |
|  | *Teruel* | 6,130 | 385 | 8,564 | 244 | 323 | 4,518 | 6,802 | 2,630 | 4,320 | 354 | 760 | 67 | 1,434 | 2,143 | 5,946 | 1,240 | 5,834 | 818 | 1,472 | 750 | 0 | 54,734 |
|  | *Zaragoza* | 16,801 | 410 | 74,191 | 673 | 4,056 | 23,600 | 65,257 | 25,057 | 26,932 | 9,457 | 7,493 | 2,487 | 17,927 | 32,974 | 26,346 | 20,299 | 39,065 | 6,788 | 12,742 | 10,060 | 10 | 422,625 |
| *ASTURIAS* | *Asturias* | 12,240 | 3,101 | 42,830 | 1,101 | 2,416 | 25,676 | 61,946 | 15,550 | 31,916 | 8,829 | 6,381 | 1,859 | 17,752 | 26,437 | 19,309 | 20,011 | 40,436 | 6,777 | 11,087 | 8,885 | 13 | 364,552 |
| *BALEARS, ILLES* | *Balearic, Islands* | 7,896 | 402 | 22,982 | 877 | 4,470 | 57,782 | 74,396 | 20,575 | 59,849 | 8,915 | 6,875 | 5,368 | 21,517 | 34,465 | 21,626 | 25,738 | 39,742 | 9,731 | 13,894 | 10,780 | 38 | 447,918 |
| *CANARY ISLANDS* | *Palmas, Las* | 12,738 | 94 | 16,124 | 697 | 3,946 | 27,231 | 84,426 | 25,976 | 81,717 | 5,071 | 5,110 | 4,189 | 17,006 | 34,468 | 22,216 | 23,430 | 37,944 | 10,360 | 13,539 | 6,658 | 56 | 432,996 |
|  | *Santa Cruz de Tenerife* | 14,408 | 125 | 13,661 | 522 | 3,831 | 25,004 | 73,989 | 19,512 | 67,068 | 5,479 | 4,662 | 4,246 | 15,988 | 29,507 | 23,779 | 21,830 | 35,106 | 8,990 | 13,136 | 5,345 | 32 | 386,220 |
| *CANTABRIA* | *Cantabria* | 7,056 | 314 | 28,393 | 555 | 1,862 | 15,895 | 34,609 | 10,117 | 18,806 | 3,090 | 3,113 | 1,060 | 9,054 | 17,080 | 12,423 | 14,458 | 22,811 | 3,463 | 6,760 | 5,520 | 4 | 216,443 |
| *CASTILE - LA MANCHA* | *Albacete* | 15,189 | 212 | 19,453 | 408 | 1,199 | 9,933 | 22,847 | 6,882 | 10,602 | 1,427 | 2,213 | 354 | 4,204 | 7,067 | 11,405 | 4,195 | 14,485 | 1,919 | 3,940 | 2,397 | 1 | 140,332 |
|  | *Ciudad Real* | 17,646 | 81 | 21,308 | 407 | 1,059 | 14,618 | 26,926 | 7,202 | 10,691 | 2,154 | 2,523 | 333 | 5,287 | 7,631 | 16,511 | 5,220 | 17,559 | 1,871 | 4,276 | 3,065 | 3 | 166,371 |
|  | *Cuenca* | 12,573 | 175 | 10,401 | 204 | 340 | 5,482 | 10,397 | 5,824 | 5,618 | 396 | 1,099 | 124 | 1,810 | 3,069 | 7,738 | 1,329 | 6,302 | 690 | 1,878 | 1,266 | 0 | 76,715 |
|  | *Guadalajara* | 3,355 | 273 | 10,725 | 533 | 661 | 7,010 | 12,646 | 11,459 | 5,952 | 657 | 1,104 | 476 | 2,555 | 9,823 | 8,121 | 2,280 | 7,957 | 1,236 | 2,518 | 1,601 | 2 | 90,944 |
|  | *Toledo* | 14,874 | 335 | 39,445 | 456 | 1,758 | 20,744 | 37,497 | 11,768 | 14,927 | 2,183 | 3,504 | 825 | 7,119 | 12,953 | 23,594 | 6,206 | 19,818 | 2,999 | 6,447 | 3,349 | 12 | 230,813 |
| *CASTILE AND LEON* | *Avila* | 5,010 | 118 | 4,663 | 131 | 393 | 4,603 | 7,920 | 1,896 | 5,219 | 355 | 1,279 | 146 | 1,548 | 2,428 | 3,818 | 3,278 | 6,814 | 637 | 1,465 | 1,164 | 1 | 52,886 |
|  | *Burgos* | 7,561 | 444 | 30,919 | 487 | 1,095 | 9,830 | 20,447 | 7,233 | 11,154 | 1,265 | 2,229 | 602 | 5,367 | 9,425 | 5,683 | 9,315 | 15,836 | 1,922 | 3,762 | 2,715 | 0 | 147,291 |
|  | *Leon* | 8,882 | 730 | 17,515 | 625 | 1,166 | 11,154 | 26,916 | 7,779 | 12,976 | 2,258 | 2,820 | 558 | 5,901 | 11,404 | 9,308 | 8,305 | 19,311 | 2,258 | 4,324 | 3,283 | 3 | 157,476 |
|  | *Palencia* | 4,870 | 71 | 12,170 | 159 | 445 | 3,445 | 8,928 | 3,050 | 4,158 | 319 | 853 | 165 | 1,776 | 5,002 | 3,725 | 2,891 | 7,918 | 709 | 1,925 | 972 | 0 | 63,551 |
|  | *Salamanca* | 7,832 | 202 | 12,055 | 266 | 1,003 | 8,438 | 19,412 | 4,355 | 10,212 | 2,093 | 1,968 | 402 | 4,850 | 7,504 | 6,725 | 8,837 | 15,305 | 2,038 | 3,497 | 2,731 | 1 | 119,726 |
|  | *Segovia* | 6,433 | 259 | 7,330 | 93 | 436 | 4,615 | 8,799 | 2,612 | 5,759 | 456 | 868 | 203 | 1,704 | 3,480 | 4,551 | 3,142 | 6,180 | 870 | 1,675 | 1,363 | 9 | 60,837 |
|  | *Soria* | 3,687 | 76 | 7,581 | 90 | 227 | 2,668 | 4,406 | 2,279 | 2,872 | 265 | 588 | 78 | 1,032 | 1,755 | 2,672 | 1,996 | 4,447 | 545 | 931 | 763 | 0 | 38,958 |
|  | *Valladolid* | 10,100 | 62 | 33,500 | 354 | 943 | 12,739 | 32,592 | 9,309 | 14,821 | 4,381 | 3,757 | 921 | 10,498 | 21,340 | 12,434 | 13,468 | 22,312 | 4,170 | 5,872 | 4,387 | 6 | 217,966 |
|  | *Zamora* | 6,846 | 129 | 5,842 | 173 | 272 | 4,526 | 9,106 | 2,322 | 4,555 | 323 | 963 | 123 | 1,937 | 2,642 | 3,838 | 2,724 | 7,209 | 589 | 1,481 | 899 | 0 | 56,499 |
| *CATALONIA* | *Barcelona* | 16,419 | 1,799 | 332,718 | 2,424 | 18,446 | 143,676 | 456,524 | 137,874 | 185,186 | 110,605 | 54,528 | 27,804 | 193,152 | 234,756 | 146,642 | 147,682 | 234,846 | 57,030 | 75,797 | 53,952 | 558 | 2,632,418 |
|  | *Girona* | 8,683 | 300 | 50,676 | 422 | 2,710 | 28,311 | 56,069 | 13,262 | 32,726 | 3,841 | 4,293 | 3,011 | 12,700 | 19,021 | 24,963 | 11,772 | 24,476 | 6,998 | 8,624 | 5,255 | 10 | 318,123 |
|  | *Lleida* | 19,467 | 232 | 25,167 | 520 | 1,461 | 14,212 | 31,174 | 10,318 | 14,270 | 2,742 | 2,514 | 755 | 7,062 | 8,933 | 16,001 | 7,948 | 16,345 | 3,756 | 4,494 | 2,277 | 2 | 189,650 |
|  | *Tarragona* | 14,140 | 351 | 42,658 | 1,305 | 3,385 | 24,188 | 47,609 | 15,090 | 24,922 | 3,650 | 3,792 | 1,994 | 14,142 | 20,356 | 25,864 | 13,510 | 27,318 | 5,243 | 8,919 | 4,099 | 8 | 302,543 |
| *CEUTA* | *Ceuta* | 250 | 5 | 403 | 102 | 477 | 1,240 | 4,483 | 1,320 | 1,809 | 206 | 169 | 60 | 521 | 1,592 | 3,980 | 805 | 2,325 | 501 | 770 | 2,182 | 0 | 23,200 |
| *VALENCIAN COMMUNITY* | *Alicante* | 22,853 | 631 | 82,616 | 620 | 5,977 | 51,575 | 133,528 | 26,976 | 74,944 | 10,793 | 11,045 | 8,988 | 27,590 | 38,332 | 30,587 | 37,570 | 53,208 | 11,639 | 20,019 | 11,110 | 64 | 660,665 |
|  | *Castellon* | 13,892 | 284 | 45,434 | 301 | 2,400 | 15,813 | 42,846 | 9,956 | 19,665 | 2,319 | 3,327 | 1,364 | 8,682 | 12,574 | 11,905 | 13,368 | 16,954 | 4,048 | 6,433 | 4,232 | 2 | 235,799 |
|  | *Valencia* | 40,751 | 566 | 138,577 | 1,819 | 8,734 | 64,611 | 202,222 | 55,039 | 79,358 | 23,768 | 17,543 | 8,336 | 54,354 | 71,073 | 45,988 | 67,527 | 83,361 | 20,180 | 27,912 | 19,579 | 100 | 1,031,398 |
| *EXTREMADURA* | *Badajoz* | 47,894 | 598 | 16,954 | 680 | 2,037 | 14,463 | 38,594 | 8,361 | 14,808 | 2,603 | 3,700 | 603 | 8,592 | 10,743 | 26,716 | 11,062 | 26,178 | 2,698 | 6,339 | 2,742 | 5 | 246,370 |
|  | *Caceres* | 25,442 | 249 | 8,929 | 784 | 1,228 | 11,383 | 19,614 | 3,902 | 10,751 | 1,292 | 1,915 | 300 | 4,794 | 6,050 | 14,003 | 7,807 | 16,090 | 1,499 | 3,605 | 2,021 | 3 | 141,661 |
| *GALICIA* | *Coruña, A* | 20,048 | 413 | 50,413 | 1,068 | 3,186 | 35,115 | 73,741 | 20,234 | 32,618 | 12,721 | 7,830 | 1,949 | 25,361 | 30,139 | 23,480 | 24,882 | 39,374 | 7,623 | 13,360 | 11,431 | 13 | 434,999 |
|  | *Lugo* | 15,873 | 388 | 12,226 | 189 | 568 | 8,691 | 20,287 | 6,713 | 9,182 | 1,557 | 1,867 | 338 | 4,283 | 5,195 | 8,466 | 5,043 | 12,419 | 1,496 | 3,149 | 3,298 | 1 | 121,229 |
|  | *Ourense* | 4,813 | 619 | 13,866 | 339 | 600 | 8,394 | 17,648 | 4,995 | 8,085 | 1,122 | 1,637 | 312 | 3,521 | 4,365 | 7,302 | 4,952 | 12,135 | 1,171 | 3,246 | 3,049 | 1 | 102,172 |
|  | *Pontevedra* | 20,480 | 484 | 59,141 | 317 | 2,204 | 24,258 | 63,344 | 17,216 | 25,476 | 5,908 | 6,185 | 1,466 | 16,023 | 23,123 | 15,186 | 19,159 | 29,511 | 5,311 | 10,690 | 8,517 | 23 | 354,022 |
| *MADRID, COMMUNITY OF* | *Madrid* | 10,705 | 1,537 | 184,044 | 7,961 | 17,507 | 189,559 | 501,683 | 181,675 | 225,985 | 240,989 | 119,597 | 33,902 | 303,115 | 338,194 | 175,549 | 214,428 | 258,326 | 63,915 | 88,875 | 119,556 | 2,307 | 3,279,409 |
| *MELILLA* | *Melilla* | 109 | 1 | 498 | 80 | 529 | 1,563 | 4,984 | 1,245 | 1,822 | 265 | 180 | 44 | 680 | 2,423 | 3,292 | 921 | 3,036 | 571 | 546 | 1,712 | 0 | 24,501 |
| *MURCIA, REGION OF* | *Murcia* | 92,620 | 698 | 71,375 | 549 | 4,845 | 37,830 | 108,155 | 26,914 | 41,988 | 8,301 | 8,080 | 2,744 | 21,417 | 31,821 | 23,342 | 31,680 | 48,805 | 8,993 | 14,825 | 11,489 | 23 | 596,494 |
| *NAVARRE* | *Navarra* | 11,823 | 310 | 67,230 | 853 | 2,362 | 17,157 | 36,606 | 12,311 | 18,155 | 3,626 | 3,918 | 878 | 13,940 | 17,118 | 11,671 | 20,703 | 29,550 | 6,110 | 7,202 | 7,377 | 13 | 288,913 |
| *BASQUE COUNTRY* | *Araba/Álava* | 3,194 | 137 | 37,972 | 151 | 759 | 7,225 | 19,010 | 7,317 | 9,689 | 2,578 | 1,713 | 434 | 7,099 | 11,294 | 12,727 | 11,686 | 16,458 | 3,166 | 4,291 | 2,983 | 4 | 159,887 |
|  | *Gipuzkoa* | 3,342 | 135 | 68,661 | 231 | 2,003 | 18,619 | 42,964 | 13,904 | 24,125 | 5,550 | 6,196 | 1,340 | 19,180 | 17,197 | 14,501 | 26,213 | 36,359 | 6,068 | 9,358 | 9,983 | 11 | 325,940 |
|  | *Vizcaya* | 5,761 | 285 | 67,721 | 1,140 | 3,495 | 31,513 | 74,488 | 21,339 | 33,915 | 15,258 | 9,881 | 2,390 | 31,242 | 36,646 | 22,407 | 38,564 | 51,736 | 8,212 | 14,730 | 16,634 | 44 | 487,401 |
| *RIOJA, LA* | *Rioja, La* | 8,737 | 141 | 25,447 | 197 | 1,020 | 8,465 | 19,387 | 4,579 | 9,932 | 1,600 | 1,992 | 493 | 4,666 | 7,006 | 6,642 | 9,069 | 11,824 | 2,301 | 3,439 | 2,772 | 7 | 129,716 |
| TOTAL |  | 1,146,818 | 23,047 | 2,073,234 | 35,809 | 145,257 | 1,261,151 | 3,207,004 | 935,898 | 1,593,479 | 576,264 | 381,062 | 147,258 | 1,064,164 | 1,430,881 | 1,111,631 | 1,098,301 | 1,723,430 | 347,107 | 539,516 | 435,745 | 3,609 | 19,280,665 |

*Source: Own preparation based on INSS^17^*

**Table A2.2 Social Security affiliated workers by age group and autonomous community (average percentage of February 2020).**

| ***Autonomous Communities*** | ***Province*** | ***From 16 to 29 years*** | ***From 30 to 39 years*** | ***From 40 to 49 years*** | ***From 50 to 59 years*** | ***More than 60 years*** | ***No data*** | ***Total*** |
| --- | --- | --- | --- | --- | --- | --- | --- | --- |
| *ANDALUSIA* | *Almeria* | 14.92% | 25.88% | 30.30% | 21.59% | 7.31% | 0.00% | 100% |
|  | *Cadiz* | 12.71% | 24.62% | 31.19% | 24.06% | 7.42% | 0.00% | 100% |
|  | *Cordoba* | 13.12% | 22.72% | 28.15% | 26.37% | 9.64% | 0.00% | 100% |
|  | *Granada* | 14.46% | 23.33% | 28.51% | 24.93% | 8.77% | 0.00% | 100% |
|  | *Huelva* | 14.51% | 25.03% | 31.65% | 22.30% | 6.51% | 0.00% | 100% |
|  | *Jaen* | 13.42% | 22.65% | 27.95% | 26.94% | 9.04% | 0.00% | 100% |
|  | *Malaga* | 13.95% | 25.08% | 30.59% | 22.95% | 7.43% | 0.00% | 100% |
|  | *Sevilla* | 14.21% | 24.75% | 31.04% | 22.97% | 7.03% | 0.00% | 100% |
| *ARAGON* | *Huesca* | 12.80% | 21.63% | 29.67% | 25.87% | 10.03% | 0.00% | 100% |
|  | *Teruel* | 12.09% | 22.44% | 28.85% | 27.37% | 9.25% | 0.00% | 100% |
|  | *Zaragoza* | 13.59% | 21.93% | 30.84% | 25.12% | 8.52% | 0.00% | 100% |
| *ASTURIAS* | *Asturias* | 9.49% | 21.73% | 32.48% | 26.20% | 10.09% | 0.00% | 100% |
| *BALEARS, ILLES* | *Balearic, Islands* | 13.77% | 24.38% | 30.80% | 23.00% | 8.05% | 0.00% | 100% |
| *CANARY ISLANDS* | *Palmas, Las* | 14.24% | 23.89% | 31.13% | 23.85% | 6.89% | 0.00% | 100% |
|  | *Santa Cruz de Tenerife* | 13.80% | 24.20% | 31.38% | 23.57% | 7.05% | 0.00% | 100% |
| *CANTABRIA* | *Cantabria* | 10.74% | 22.67% | 31.64% | 25.35% | 9.59% | 0.00% | 100% |
| *CASTILE - LA MANCHA* | *Albacete* | 13.54% | 22.92% | 29.33% | 25.90% | 8.32% | 0.00% | 100% |
|  | *Ciudad Real* | 13.80% | 24.18% | 29.03% | 24.94% | 8.05% | 0.00% | 100% |
|  | *Cuenca* | 13.42% | 22.43% | 28.81% | 26.69% | 8.66% | 0.00% | 100% |
|  | *Guadalajara* | 12.85% | 23.98% | 31.20% | 24.31% | 7.66% | 0.00% | 100% |
|  | *Toledo* | 12.42% | 23.92% | 31.36% | 24.24% | 8.06% | 0.00% | 100% |
| *CASTILE AND LEON* | *Avila* | 9.80% | 20.05% | 29.24% | 29.18% | 11.73% | 0.00% | 100% |
|  | *Burgos* | 11.02% | 21.11% | 30.10% | 27.46% | 10.31% | 0.00% | 100% |
|  | *Leon* | 10.46% | 21.58% | 29.65% | 27.78% | 10.52% | 0.00% | 100% |
|  | *Palencia* | 10.52% | 21.82% | 28.27% | 27.88% | 11.50% | 0.00% | 100% |
|  | *Salamanca* | 12.23% | 20.87% | 28.08% | 28.11% | 10.70% | 0.00% | 100% |
|  | *Segovia* | 11.25% | 20.27% | 28.70% | 28.86% | 10.92% | 0.00% | 100% |
|  | *Soria* | 11.51% | 20.30% | 28.44% | 28.93% | 10.83% | 0.00% | 100% |
|  | *Valladolid* | 12.40% | 21.52% | 31.18% | 25.80% | 9.09% | 0.00% | 100% |
|  | *Zamora* | 9.09% | 20.11% | 28.50% | 29.93% | 12.37% | 0.00% | 100% |
| *CATALONIA* | *CATALONIA* | 16.04% | 22.99% | 30.57% | 22.84% | 7.56% | 0.00% | 100% |
| *CEUTA* | *Ceuta* | 11.83% | 23.02% | 29.35% | 26.11% | 9.68% | 0.00% | 100% |
| *VALENCIAN COMMUNITY* | *Alicante* | 13.32% | 23.47% | 31.09% | 24.12% | 8.01% | 0.00% | 100% |
|  | *Castellon* | 13.38% | 22.63% | 32.12% | 24.46% | 7.41% | 0.00% | 100% |
|  | *Valencia* | 14.29% | 22.90% | 31.49% | 23.81% | 7.51% | 0.00% | 100% |
| *EXTREMADURA* | *Badajoz* | 13.13% | 23.75% | 29.09% | 25.58% | 8.44% | 0.00% | 100% |
|  | *Caceres* | 11.07% | 22.33% | 27.91% | 28.43% | 10.26% | 0.00% | 100% |
| *GALICIA* | *GALICIA* | 11.12% | 22.89% | 31.87% | 25.06% | 9.06% | 0.00% | 100% |
| *MADRID, COMMUNITY OF* | *Madrid* | 16.27% | 24.47% | 30.06% | 22.09% | 7.11% | 0.00% | 100% |
| *MELILLA* | *Melilla* | 15.47% | 24.17% | 27.32% | 24.05% | 8.99% | 0.00% | 100% |
| *MURCIA, REGION OF* | *Murcia* | 14.74% | 24.56% | 31.04% | 22.51% | 7.15% | 0.00% | 100% |
| *NAVARRE* | *Navarra* | 13.78% | 21.89% | 31.06% | 25.32% | 7.96% | 0.00% | 100% |
| *BASQUE COUNTRY* | *Araba/Álava* | 12.67% | 22.16% | 31.43% | 26.01% | 7.73% | 0.00% | 100% |
|  | *Gipuzkoa* | 12.39% | 20.50% | 31.51% | 26.73% | 8.87% | 0.00% | 100% |
|  | *Vizcaya* | 11.03% | 21.10% | 31.72% | 27.15% | 9.01% | 0.00% | 100% |
| *RIOJA, LA* | *Rioja, La* | 12.62% | 21.87% | 30.88% | 25.53% | 9.09% | 0.00% | 100% |
| *TOTAL* |  | 14.13% | 23.36% | 30.62% | 23.94% | 7.95% | 0.00% | 100% |

*Source: Own preparation based on TGSS^18^*
