# Supplementary material for: Who can go back to work when the COVID-19 pandemic remits?
Source: PLoS One. 2020 Aug 27;15(8):e0238299. doi: 10.1371/journal.pone.0238299 (PMC7451540; doi:10.1371/journal.pone.0238299)
Supplement: Attachment — Submitted filename: ResponsetoReviewers.docx [file pone.0238299.s003.docx]

Dear PLOS ONE reviewer,

We are extremely grateful for your comments on our article “Who can go back to work when the COVID-19 pandemic subsides?”. Please take this letter as an explanation of the changes that we have sought to implement in our work, considering all your suggestions.

We have carefully read the comments made in your report and we have considered how to include your suggestions. We agree with you on the need to highlight the document that you have indicated to us, and we have done so. In this regard, we wished to point out that our article was first completed on a date when the Baqaee, Farhi, Mina and Stock research was not yet accessible (our document was published on May 10 in a preprint version).

Likewise, as you point out, in the version of our article that we are sending now we have included a reflection on the limitation that contagion in non-work activities implies developing a lockdown de-escalation strategy (P.17). The new version of the article that we are sending now also includes the results of the research of Baqaee et al. (2020) (P. 15).

Based on the above, we hope that this new revised version of our article successfully addresses the shortcomings you pointed out in the previous version and that it now meets the requirements for publication in the journal. Please do not hesitate to contact us should you wish to clarify any other question related to our work.

One again, we are grateful for your comments, as these have enabled us to substantially improve our article.

Kind regards.

The authors.
